# Editorial on Anticancer Antioxidants

**DOI:** 10.3390/antiox10111782

**Published:** 2021-11-08

**Authors:** Suzana Borovic Sunjic, Neven Zarkovic

**Affiliations:** Laboratory for Oxidative Stress, Division of Molecular Medicine, Rudjer Boskovic Institute, Bijenicka 54, 10000 Zagreb, Croatia; borovic@irb.hr

The current concepts of biomedicine consider oxidative stress to be one of crucial pathophysiological processes behind major stress- and age-associated diseases, including cancer. Consequently, antioxidants are frequently believed to be an almost universal defense that could prevent or even cure malignant diseases. However, the study conducted in the 1990s caused harm not only to the people involved but also to research on anticancer antioxidants. Namely, the ATBC study exposed volunteers to an overload (several fold above controls) of lipid-soluble antioxidants, which accumulate in biomembranes and could become dangerous in case of prolonged exposure to the carcinogenic stressors of cigarette smoking for the volunteers involved in the trial [1].

However, many anticancer treatments rely on the cytotoxic effects of ROS, even if this affects non-malignant cells, while differences in the antioxidant mechanisms between cancer cells and their counterpart non-malignant cells are not well-understood. Among these, oxidative lipid metabolism, especially the non-enzymatic, self-catalysed peroxidation of poly-unsaturated fatty acids (PUFAs), is important for carcinogenesis, as well as for cancer growth control and anticancer treatments [2,3,4]. Therefore, this Special Issue of *Antioxidants* collected original research papers and reviews on the complex biomedical effects of pro- and anti-oxidants that affect cancer growth control. Due to the tough selection process for submitted papers that was carried out by the editorial office of the journal, the majority of submitted papers were not published in this Special Issue, but we hope that the further progress in the field will include their contributions.

The relevance of iron, considered a necessary co-factor of cellular growth, with strong pro-oxidant activities, was studied in a clinical trial performed by researchers from the famous German Cancer Research Center in Heidelberg, who found that ferritin, transferrin and C-reactive protein (CRP) levels are not associated with colorectal adenoma in obese patients [5]. However, they suggested that high transferrin saturation could reflect the organism’s iron overload, while a low concentration of total thiol groups (-S-H) of serum proteins may reflect systemic redox imbalance in obese patients. This could, at least partially, explain the associations of iron overload and obesity with colorectal adenoma, supporting the previously described association of iron accumulation and lipid peroxidation in colon carcinogenesis [6,7]. On the other hand, iron might be also considered a potentially beneficial anticancer pro-oxidant that triggers the peroxidation of PUFAs, thus generating cytotoxic and growth-regulating aldehydes, especially 4-hydroxynonenal (HNE) [8].

The other original research papers in this Special Issue present the results of in vitro and in vivo research tackling molecular mechanisms of possible anticancer effects of particular antioxidants, as well as the cellular and systemic defense mechanisms relevant for cancer growth control. Thus, Romanian research studied the in vitro effects of the lichen Usnea barbata (L.) F.H. Wigg. (U. barbata), by comparing their effects on malignant and non-malignant cells [9]. The relatively selective anticancer effects of these extracts were mostly attributed to an imbalance in antioxidant defense mechanisms, causing oxidative stress in cancer cells, thus resembling the previously mentioned effects of iron and HNE. Scientists from Taiwan revealed in vitro that withaferin A (WFA), the Indian ginseng bioactive compound, exhibits anticancer effects against bladder cancer cells through the induction of oxidative stress [10]. These pro-oxidative and anticancer effects of WFA were attenuated by the antioxidant N-acetylcysteine, which is also known to be a very efficient scavenger of HNE [11]. Aiming to evaluate the molecular mechanisms of possible anticancer effects of the well-known antioxidant astaxanthin (ATX), Korean researchers studied its effects in vitro and in vivo on myeloid-derived suppressor cells (MDSCs), particularly their cell-signaling pathways, including nuclear factor erythroid-derived 2-related factor 2 (Nrf2) [12]. They observed that, upon ATX treatment, functional mediators of immune suppression were significantly reduced, while the antioxidant activity of ATX reduced oxidative stress in MDSCs, which became immunogenic enough to induce cytotoxic T lymphocyte response and an inhibition of tumor growth. The signaling pathway of Nrf2 was also the focus of research by an international European team analyzing how Nrf2 maintains the expression of its targets under homeostatic conditions in lung cancer cells [13]. They described the sTable 105 kDa Nrf2 form, which is resistant to Keap1-Cul3-mediated degradation and translocates to the nucleus of lung cancer cells. This sTable 105 kDa Nrf2 form might originate from the exon 2 or exon 3-truncated transcripts, while further studies could help to explain the constitutive activity of Nrf2 under normal cellular conditions, i.e., without oxidative stress.

The review paper on Nrf2 signaling, written by Croatian scientists, focused mostly on its specific targets, including those involved in thioredoxin (TRX) and glutathione (GSH) systems [14]. Summarizing data collected from more than three hundred research and review papers, these scientists concluded that targeting only one signaling system controlled by Nrf2 can be beneficial, while the combined modulation of multiple antioxidant systems can provide better anticancer results. Therefore, miRs modulating the NRF2 pathway and TRX and GSH antioxidant system efficiencies should also be considered in anticancer therapies, thus supporting previous research and reviews in the field, especially those tacking controversies in the field and Nrf2’s relationship with HNE and lipid peroxidation [15,16,17]. Another review paper on Nrf2 signaling was written by Canadian scientists who summarized the dual effects of flavonoids in cancer prevention and cancer promotion based on the regulation of the Nrf2/ARE pathway [18]. They wrote that the most popular flavonoids can activate the Nrf2/ARE pathway in both normal and cancer cells, while their hormetic effects reflect their concentration-dependent anti-oxidant or pro-oxidant activities. Therefore, some flavonoids can enhance cancer growth and suppress the Nrf2/ARE pathway, suggesting a need for thorough further research.

The American group of scientists wrote a review on dietary polyphenols’ role in the prevention of pancreatic cancer, stressing the fact that herbal dietary agents possess medicinal properties, which formed the basis for their traditional use to treat various diseases, relying on their anti-inflammatory and antioxidant properties, particularly in the case of their anticancer activities [19]. Focusing especially on the most popular, quercetin and resveratrol, these researchers summarized findings of in vitro and animal studies that indicate the potential of these polyphenols to enhance the efficacy of standard chemotherapeutic agents and other natural compounds against experimental pancreatic cancer, providing a rationale for their exploration in clinical trials. However, they also point to a lack of common knowledge on the safety of these polyphenols if used during cancer treatment and request extensive bioinformatics to predict the targets of the bioactivities of these polyphenols. Complementary to this review, a Canadian group of researchers from Ontario prepared a review on a particular naturally occurring phyto-polyphenol (diterpene) carnosol found in rosemary, which has been studied for its antioxidant, anti-inflammatory and anticancer effects, inhibiting the proliferation and survival of malignant cells, reducing their migration and invasion, and enhancing apoptosis [20]. The inhibition of several signalling molecules, including extracellular signal-regulated kinase (ERK), p38, c-Jun N-terminal kinase (JNK), Akt, mechanistic target of rapamycin (mTOR) and cyclooxygenase-2 (COX-2), seem to be important targets for the anticancer effects of carnosol, which also prevents the nuclear translocation of NF-κB. In spite of the detailed knowledge gained of the molecular activity principles of carnosol, the authors conclude that further studies are required to fully understand the effects of carnosol in both cancerous and normal tissues, particularly lung, colon, and pancreatic malignancies.

Researchers from South Africa/India wrote a review with a quite general title on the redox potential of antioxidants in cancer progression and prevention, mainly focusing on a metabolic reprogramming arising from mutations in the metabolic enzymes of cancer cells that can lead to the overproduction of so-called ’oncometabolites’ in a state of ‘pseudohypoxia’. They offered photodynamic therapy (PDT) using light-activated photosensitizing molecules that can regulate cellular redox balance in accordance with the changes in endogenous ROS production as a possible solution to many challenges in cancer therapy [21]. In conclusion, the authors state that the supplementation of vitamin C protects healthy cells against the damage caused by excessive oxidative stress and the risk of cancer and cardiovascular disorders due to cigarette smoking [22], assuming it is suitable to stick to selected antioxidants as a form of dietary supplementation. Vitamin C was also of interest to an international Arabian team of scientists, who wrote a review on the liposomal delivery of ascorbic acid and alpha-lipoic acid enhancing their antioxidant and anticancer effects using liposomes as nanocarriers [23]. As it is likely the most popular water-soluble antioxidant and enzyme co-factor produced by plants and certain animals, vitamin C is an essential micronutrient antioxidant, with a powerful reducing effect, that plays important roles in numerous physiological processes in the human body. Similar to ascorbic acid, alpha-lipoic acid is also naturally occurring, but it is a short-chain fatty acid, which contains sulphur to support its use as an antioxidant, a nutritional supplement and an adjuvant medicinal remedy within the integrative biomedicine. The authors of this review recommend liposomes as promising nanocarriers to deliver effective concentrations of antioxidants to the cancer tissue, in addition to the confirmed synergistic effects of antioxidants with traditional anticancer agents.

Finally, O. Cauli from Valencia, Spain wrote a review on oxidative stress and cognitive alterations induced by cancer chemotherapy drugs [24]. Namely, chemotherapy-based regimens such as anthracyclines, doxorubicin, taxanes and platinum derivatives can induce both oxidative stress in the blood and in the brain, leading to cognitive impairment, which is one of the most deleterious effects of chemotherapy treatment in cancer patients and can persist even after chemotherapy ends. The author gave arguments in favor of the use of antioxidants such as N-acetylcysteine, gamma-glutamyl cysteine ethyl ester, polydatin, caffeic acid phenethyl ester and 2-mercaptoethane sulfonate sodium (MESNA) to counteract both the oxidative stress and cognitive alterations induced by chemotherapeutic drugs. It should be mentioned that some of these antioxidants were proposed for application as integral parts of treatment protocols against COVID-19, which was recently found to be associated with systemic vascular lipid peroxidation [25]. Moreover, a major bioactive marker of lipid peroxidation, the reactive aldehyde HNE described at the beginning of this Editorial, was recently revealed to be associated with post-traumatic stress disorder (PTSD), persisting for decades after acute stress [26], and the onset of brain tumors [27].

In conclusion, one may say that at present, unlike the premature, badly designed and consequently harmful clinical trials on anticancer antioxidants performed thirty years ago, modern scientists are more carefully approaching this complex topic, considering every patient as an individual and targeting cancer as malignant condition of persistent oxidative stress, thus providing arguments in favor of antioxidants as integral components of modern integrative biomedicine of cancer and other oxidative-stress-associated disorders.
